# Patriarchy, Fertility and Excess Female Child Mortality in India

**DOI:** 10.1007/s40980-024-00133-z

**Published:** 2025-01-09

**Authors:** Abhishek Singh, Kaushalendra Kumar, Lotus McDougal, Praveen Kumar Chokhandre, Ajeet Kumar Singh, Ashish Kumar Upadhyay, K. S. James, Anita Raj

**Affiliations:** 1 Department of Public Health and Mortality Studies, International Institute for Population Sciences, Mumbai, India; 2 Centre of Demography of Gender, International Institute for Population Sciences, Mumbai, India; 3 Center on Gender Equity and Health, University of California San Diego, San Diego, USA; 4 Population Research Centre, JSS Institute of Economic Research, Dharwad, India; 5 Immunization Technical Support Unit, Ministry of Health & Family Welfare, New Delhi, India; 6 GENDER Project, International Institute for Population Sciences, Mumbai, India; 7 Newcomb Institute, Tulane University, New Orleans, LA USA; 8 Tulane School of Public Health and Tropical Medicine, New Orleans, LA USA

**Keywords:** Patriarchy, India patriarchy index, TFR, Excess female child mortality, India

## Abstract

Research investigating association between patriarchy and demographic behavior is limited in India. The only study on this subject utilized 1981 Indian Census data to examine associations between patriarchy and fertility. We examined the association of patriarchy, measured using India Patriarchy Index (IPI), with total fertility rate (TFR) and excess female child mortality in India. Additionally, we examined independent associations of the 5 dimensions included in the IPI with the two outcomes. We used univariate and bivariate Local Indicators of Spatial Autocorrelation, multivariable ordinary least squares and spatial error- regressions to examine the associations. Spatial heterogeneity beyond the north–south divide was evident in the spatial association of IPI with TFR and excess female child mortality. Results show positive association of IPI with TFR and excess female child mortality. While son preference and socio-economic domination were positively associated with TFR, domination of men over women and son preference were positively associated with excess female child mortality. This study is the first of its kind to examine the association of a novel measure of patriarchy with TFR and excess female child mortality. As patriarchy is deep-rooted in Indian society, a great deal of effort is needed to shift these traditionally held social norms and practices.

## Introduction

Patriarchy is the socio-structural system promoting and enabling male control over women by means of domination, oppression and exploitation (Walby, 1989). Patriarchal norms contribute to gender inequality by giving men priority over women in access to resources in the family, market, state, and society, writ large (Walby, 1989). India is a country where patriarchal norms are deeply rooted and practiced in different forms since ancient times. While patriarchy prevails throughout India, the magnitude of patriarchal norms and practices varies considerably across states, urban–rural residence, and communities (Agarwal, 1994; Bagchi, 1981; Chakraborty & Kim, 2010; Karve, 1965; Nasir & Kalla, 2006; Singh et al., 2021).

Patriarchal norms manifest in multiple ways in India, such as son preference, regressive gender norms, compromised women’s empowerment, kinship system that is in favor of males, etc. Patriarchal norms, through their multiple facets, affect a number of demographic outcomes, such as higher fertility, high infant and child mortality, excess female infant and child mortality, and lower contraceptive use (Arokiasamy, 2002; Bhattacharya, 2006; Bose & Das, 2024; Chaudhuri, 2012; Chaudhuri, 2015; Clark, 2000; Das Gupta 2003; Guilmoto et al., 2018; Haque et al., 2021; Kashyap, 2019; Kashyap & Behrman, 2020; Malhotra et al., 1995). These studies have linked different facets of patriarchy with demographic outcomes in isolation. While it is important to understand different facets of patriarchy and their association with demographic outcomes in isolation, understanding patriarchy per se and its association with demographic outcomes is all the more important. A comprehensive measure of patriarchy may afford better understanding of the gender inequality present in the Indian population. Such a measure may also aid in examining the trends in gender inequality over time. In this study we use India Patriarchy Index (IPI), developed by Singh et al. (2021) for measuring patriarchy in India and its states and districts, to examine association of patriarchy with fertility and excess female child mortality at the district-level. We hypothesize that high patriarchy will be associated with a) high TFR and b) high excess female child mortality. We then examine whether accounting for spatial clustering modifies the relationship of patriarchy with TFR and excess female child mortality. We also examine whether the spatial distribution of patriarchy and the spatial distribution of the two dependent variables are correlated at the district level. Finally, we conducted secondary analyses in which we examined independent associations of the 5 dimensions included in the IPI with the two outcomes. This work extends prior research by using a more comprehensive and validated measure of patriarchy (i.e. IPI) and focus on outcomes that demonstrate ongoing gender inequalities in India.

## Research Context

Patriarchal norms could affect demographic outcomes at various levels. Patriarchal norms could restrict women’s choices and opportunities (Doss, 2013; Kabeer, 2005), such as when and whom to marry, access to education, paid work and other financial services, participation in civil society and political institutions, etc., leading to adverse demographic outcomes. Such restrictions may compromise women’s control over their fertility decisions. Indian studies have shown how parents use different stopping rules to have the desired number of male children (Basu & De Jong, 2010; Bongaarts, 2013; Clark, 2000; Chaudhuri, 2012), thereby increasing fertility. Patriarchal norms may also lead to unequal allocation of food, preventive and curative care, and other household resources among male and female children in India (Barcellos et al., 2014; Fledderjohann et al., 2014; Jayachandran & Kuziemko, 2011; Mishra et al., 2004; Pande, 2003; Pande & Yazbeck, 2003; Rajan & Morgan, 2018). Such unequal allocation of household resources may lead to excess female child mortality in India. Apart from the characteristics of the household members, household-level patriarchal norms are often shaped by the immediate community and broader society. Each household’s patriarchal norms may vary by the degree of adherence or resistance to the community or broader society’s norms (Scott et al., 2021). Therefore, patriarchal norms at community- or broader society-level may also affect fertility and excess female child mortality.

India is a large and diverse country comprising of 28 states and 8 Union Territories (UTs). Patriarchal norms and demographic outcomes often vary a lot across the different states and UTs of India. There is a clear north–south divide when it comes to fertility, mortality, son preference, women’s autonomy and decision-making, etc. (Bharati et al. 2011; Dyson & Moore, 1983; Guilmoto et al., 2018; Radkar, 2018; Singh et al., 2021). Indian studies have also shown strong spatial clustering of son preference, fertility and excess female child mortality across the districts of India (Guilmoto et al., 2018; Singh et al., 2017; Singh et al. 2021). Singh et al. (2021) showed strong spatial clustering of IPI across the Indian districts. These studies call for sub-state level analysis to capture spatial heterogeneity in associations of patriarchal norms with fertility and excess female child mortality; the finer the level of observation, the greater the degree of accuracy in identifying the spatial associations. Furthermore, examining such associations may help in identifying subnational clusters where progress is lagging and offering valuable planning aids to policy-makers in designing interventions that account for the local context.

A number of studies from India have examined association of various facets of patriarchal norms and fertility in India (Bhattacharya, 2006; Bose & Das, 2024; Chaudhuri, 2012; Haque et al., 2021; Jayaraman et al., 2009; Malhotra et al., 1995; Singh et al., 2017). While some studies found positive association between gender inequality and fertility (Bhattacharya, 2006; Chaudhuri, 2012; Haque et al., 2021; Jayaraman et al., 2009; Malhotra et al., 1995; Singh et al., 2017), others reported inconsistent or no relationship (Bose & Das, 2024; Moursund & Kravdal, 2003). A few studies have also examined association between patriarchal norms and excess female child mortality in India (Arokiasamy, 2004; Bhalotra & Cochrane, 2010; Bhattacharya, 2006; Chaudhuri, 2015; Guilmoto et al., 2018; Kashyap, 2019; Kashyap & Behrman, 2020; Rosenblum, 2013). All these studies reported positive association between gender inequality and excess female child mortality in India.

Malhotra et al. (1995) measured three overlapping and multidimensional aspects of patriarchy—active discrimination, marriage system, and economic value of women—using six variables (the sex ratio of mortality, the female share of literates, the proportion of females aged 15–19 unmarried, excess female migration, the female share of the labor force, and the area under rice cultivation)—and examined the independent association of three dimensions with fertility. They concluded that patriarchy plays an important role in explaining regional variations in fertility in India. We could not come across any Indian studies that examined association between patriarchy and excess female child mortality.

While the existing literature have added to the debate on patriarchy and demographic outcome in India, defining and measuring patriarchy per say and examining association of a comprehensive measure of patriarchy with fertility and excess female child mortality is an important missing link in the Indian demographic literature. In addition, none of the existing studies have examined association of a comprehensive measure of patriarchy with fertility and excess female child mortality at more local levels, such as districts. An important development to address this missing link in the Indian literature is the India Patriarchy Index (IPI) (Singh et al., 2021)^1^. Singh et al. (2021) included demographic indicators on marital age and practices, family structure and roles by age, sex of offspring and power relations within the household, educational hypogamy marriages, and women’s work to construct IPI. Singh et al. (2021) used data from the 2015–16 National Family Health Survey (NFHS) conducted in 640 districts across India in 2015–16 (henceforth known as NFHS-4). They included 5 dimensions in the IPI–1) domination of men over women, 2) domination of older generation over the younger generation, 3) patrilocality, 4) son preference, and 5) socio-economic domination—directly capturing the key defining features of familial patriarchy in India (Singh et al., 2021). A key defining feature of family patriarchy is the domination of men over women in the family or household. This dimension, in IPI, is captured through three variables – wives as household head, young brides and older wives. An equally important dimension of family patriarchy is the domination of older generation over the younger generation. This dimension, in IPI, is captured through three variables—younger household head, neo-local residence and joint families. Patrilocality, an important defining feature of family patriarchy is measured by married daughters. Son preference another important definition feature of family patriarchy is captured through three variables – boy as last child, sex ratio and ideal number of sons. Finally, the fifth dimension, socio-economic domination, was captured through two variables—educated wives and economic domination. The rationale for including the above five dimensions and other details of IPI may be found in Singh et al. (2021).

Singh et al. (2021) showed that the IPI is reliable and has high construct validity for measuring patriarchy at state and lower administrative levels, such as districts, in India. Additionally, Singh et al. (2021) showed that the distribution of patriarchy (measured using the IPI) over geographies and communities (in terms of caste, religion, urban–rural residence, and land ownership) corresponds very well with the Indian sociological literature on patriarchy.

A clear advantage of the IPI over other widely used indices of gender inequality, such as Gender Inequality Index (GII) and Social Institutions and Gender Index (SIGI), is that IPI can be easily computed using the Demographic and Health Surveys (DHS), which are conducted in over 90 low- and middle- income countries (LMICs). As DHS is frequently conducted, IPI can be used to monitor trends in gender inequality at lower administrative levels, such as districts in India. Unlike other indicators of gender inequality, which are odd combination of gender differentials in health, education, earned income, share of parliamentary seats, etc., IPI combines 5 dimensions of family patriarchy to arrive at a comprehensive index of gender inequality. Recognizing the several advantages of IPI over other existing indices, we use IPI values computed by Singh et al. (2021) and spatial analyses tools such as Moran’s I, Local Indicator of Spatial Association (LISA), ordinary least squares (OLS) regression, and spatial error regression to fulfill the objectives of our paper.

## Data and Methods

### Data

We utilized data from multiple sources to understand the association of IPI with TFR and excess female child mortality. We used the IPI for the 640 districts of India as calculated by Singh et al. (2021). District-level estimates of TFR were derived from Singh et al. (2017), who used 2011 census data—total number of births in the preceding year and the total number of women in the 5-year age groups—to calculate district-level estimates of TFR. We used excess female child mortality as calculated by Guilmoto et al. (2018), who derived district-level estimates of U5MR by sex from 2011 census data.

NFHS-4 was conducted across the 640 districts of India. The key objectives of NFHS-4 were to provide national- and state- level estimates of fertility, mortality, family planning, maternal and child health indicators, sexual life, domestic violence, HIV/AIDS, etc. (International Institute for Population Sciences (IIPS) and ICF 2017). NFHS-4, for the first time in the NFHS series, also provided select key indicators for the 640 districts of India. NFHS-4 interviewed 699,686 women age 15–49 in a total of 601,509 households. The eligible women response rate was 97% (International Institute for Population Sciences and ICF 2017).

### Dependent Variables

The dependent variables included in this study are TFR and excess female child mortality, defined in Table 1.

**Table 1:** Variable descriptions, means (SD), and Moran's I, Indian districts

Variable	Description	Mean (SD)	Minimum, Maximum value	Moran's I
*Dependent variables*				
Excess female child mortality(1)	Difference of probability of under-5 female- and male- child death per 1000 live births	15.8 (11.3)	− 17.2, 52.8	0.59
Total fertility rate (TFR)(2)	Total fertility rate	3.3 (1.0)	1.6, 5.8	0.55
*Independent variable*				
India Patriarchy index (IPI)(5)	India patriarchy index	30.6 (3.2)	14.5, 36.6	0.57
*Control variables*				
Child mortality rate(6)	Probability of dying before sixth birthday per thousand live births	61.5 (23.6)	11.0, 121.9	0.77
Contraceptive prevalence rate(4)	Percentage of currently married women using any method of contraception	50.7 (17.1)	2.7, 84.8	0.52
Childlessness(3)	Percentage of childless women age 45–49	6.2 (2.2)	0.0, 22.1	0.45
Female workforce participation(3)	Percentage of women age 15–49 employed	23.8 (13.3)	4.8, 60.0	0.53
Female secondary or higher education(4)	Percentage of women age 15 or older who have completed secondary or more education	48.4 (12.9)	16.7, 85.9	0.52
Exposure to media(4)	Percentage of women age 15–49 exposed to media	79.9 (17.17)	31.3, 99.8	0.52
Poor household(7)	Percentage of households in poorest or poorer wealth quintile	20.0 (14.7)	0.2, 66.5	0.61
Scheduled castes/tribes(3)	Percentage of scheduled caste and scheduled tribe population	32.5 (22.5)	0.3, 98.6	0.68
Hindu(3)	Percentage of hindu population	74.0 (26.8)	0.9, 99.4	0.54
Muslim(3)	Percentage of muslim population	12.9 (17.5)	0.2, 98.5	0.46
Sikh(3)	Percentage of Sikh population	2.4 (11.1)	0.0, 93.3	0.71
Percentage urban(3)	Percentage of population living in urban areas	26.4 (21.1)	0.0, 100	0.28

All the variables are calculated at the district level; Standard deviation (SD) is in parenthesis; Moran's I range between − 1 (perfect dispersion) and + 1 (perfect clustering) and 0 indicates random spatial pattern. Sources:(1) Guilmoto et al. (2018); (2) Authors’ own calculation using the total number of births in the preceding year and the total number of women in the 5-year age groups (given in Table F9) collected in Census 2011. As suggested by Bhat et al. (1984) and outlined by Vosti and Lipton (1991), we adjusted the estimated TFRs by an inflation factor that accounted for the age structure of the childbearing population and child mortality. See Singh et al. (2017) for calculation of inflation factor. While, the TFRs for five districts namely Kupwara (in Jammu & Kashmir), Budgam (in Jammu & Kashmir), Mewat (in Haryana), Jaintia Hills (in Meghalaya) and West Khasi Hills (in Meghalaya) were extremely high, the TFR for Thrissur (in Kerala) was very low (0.1). The TFRs for these districts were replaced by their state average TFRs; (3) Authors’ own calculation using Census 2011; (4) Authors’ own calculation using Indian DHS round-4 (NFHS4 2015–16) micro data; (5) Singh et al. (2021); (6) Guilmoto and Rajan (2013); (7) Authors’ own calculation using housing micro data from Census 2011

### Independent Variables

The independent variable is the district-level IPI. In total, 12 variables across the five domains were included in the construction of IPI (Singh et al., 2021). The dimension of domination of men over women included three variables—female household head, young brides, and older wives. The dimension of domination of older generation over the younger generation included three variables—younger household head, neo-local residence, and joint family. The dimension of patrilocality included one variable—married daughter. The dimension of son preference included three variables, namely boys as last child, sex ratio in children age 0–6 years, and proportion of women age 15–49 who report higher ideal number of sons than daughters. The dimension of socio-economic domination included two variables—educated wives and economic domination. Higher IPI values indicate higher patriarchy. Details of the variables used in the computation of IPI are given in Table 2. For the secondary analyses, the 5 dimensions of IPI acted as the independent variables.

**Table 2:** Description of variables employed in computation of the India Patriarchy index

Variable	Description	Definition
*Male domination*		
Female heads	Proportion of female headed household in a district	$=\frac{Number\, of \,female\, headed \,households \,in \,a \,district }{Total\, number\, of \,households\, in \,the\, district}$
Young brides	Proportion of young brides (15–19 year) in a district	$$=\frac{\begin{array}{c}Total\, number\, of\, ever\, married\, \\ young\, brides\, aged \,15-19\, year \,in\, a\, district\end{array}}{\begin{array}{c}Tota\,l \,number\, of\, ever\, married\, women \,age \\ 15-49 \,\,in\, the \,district\end{array}}$$
Older wives	Proportion of wives who are older than their husbands in a district	$$=\frac{\begin{array}{c}Total\, number \,of\\ wives \,who \,are \,older \,than \,the \,husband \,in \,a \,district \end{array}}{Total \,no. \,of \,couple \,in \,district}$$
*Generational domination*		
Younger household head	Proportion of elderly men co-residing with a younger household head	$$\frac{{\begin{array}{*{20}c} {Total\,number\,of\,elederly\,men\,living\,in\,a\,household\,headed} \\ {by\,a\,male\,of\,a\,younger\,generation\,in\,a\,district} \\ \end{array} }}{{Total\,number\,of\,elederly\,having\,at\,least\,one\,child}}$$
Neo-local	Proportion of neo-local residence among young men in a district	$$= \frac{{\begin{array}{*{20}c} {Total\,number\,of\,ever\,married\,men\,household\,head\,age\,20 - 29} \\ \end{array} }}{{Total\,number\,of\,ever\,married\,men\,age\,20{\text{ }} - {\text{ }}29}}$$
Joint family	Proportion of elderly people living in joint residence in a district	$$= \frac{{\begin{array}{*{20}c} {Total\,number\,of\,elderly\,person\,\left( {aged\,60 + } \right)\,living\,with\,at} \\ {least\,two} \\ {married\,sons\,in\,the\,same\,household\,in\,the\,district} \\ \end{array} }}{{Total\,number\,of\,elderly\,person\,\left( {aged\,60 + } \right)\,in\,the\,district}}$$
Patrilocality		
Married daughter	Proportion of elderly people living with married daughters in a district	$$= \frac{{\begin{array}{*{20}c} {Total\,number\,of\,elderly\,in\,a\,district\,\left( {aged\,60 + } \right)\,living\,with} \\ {at\,least\,one\,married\,daughter} \\ \end{array} }}{{\begin{array}{*{20}c} {Total\,number\,of\,elderly\,in\,a\,district\,\left( {age\,60 + } \right)\,living\,with\,at\,least} \\ {one\,married\,child\,in\,the\,same} \\ {household} \\ \end{array} }}$$
*Son preference*		
Boy as last child	Proportion of last birth as a boy in a district	$$= \frac{{\begin{array}{*{20}c} {Total\, number\, of\, boys\, among\, last\, children\, in\, a\, district} \\ \end{array} }}{{Total\, number\, of\, last\, birth\, in\, a\, district}}$$
Sex ratio	Sex ratio among children in the age 0–6 years in a district	$$= \frac{{\begin{array}{*{20}c} {Total\, number\, of\, male\, children\, age\, \left( {0 - 6yrs.} \right)\, in\, a\, district*100} \\ \end{array} }}{{Total\, number\, of\, female\, children\, aged\, \left( {0{\text{ }} - {\text{ }}6{\text{ }}yrs.} \right)\, in\, a\, district}}$$
Ideal no. of sons	Percent of women age 15–49 whose desire for ideal number of son is more than ideal number of daughters	$$= \frac{{\begin{array}{*{20}c} {Total\, number\, of\, women\, age\, 15 - 49\, who\, reported\, desire\, for} \\ {higher\, number\, ideal\, number\, of\, son} \\ {than\, daughter} \\ \end{array} }}{{Total\, number\, of\, women\, age\, 15{\text{ }} - {\text{ }}49}}$$
*Socio-economic domination*		
Educated wives	Proportion of wives who are more educated than their husbands in a district	$$= \frac{{\begin{array}{*{20}c} {Total\, number\, of\, wives\, who\, have\, more\, years\, of\, schooling} \\ {than\, husband} \\ \end{array} }}{{\begin{array}{*{20}c} {Total\, number\, of\, couples\, for\, w\hom e\, the\, years\, of} \\ {schooling\, are\, known\, in\, a\, district} \\ \end{array} }}$$
Economic domination	Proportion of women age 15–49 who are engaged in professional work in a district	$$= \frac{{\begin{array}{*{20}c} {Total\, number\, of\, women\, age\, 15 - 49\, working\, as} \\ {professional\, work\, in\, a\, district} \\ \end{array} }}{{Total\, number\, of\, women\, age\, 15 - 49\, year\, in\, the\, district}}$$

Source: Singh, A., Chokhandre, P., Singh, A.K. et al*.* Development of the India Patriarchy Index: Validation and Testing of Temporal and Spatial Patterning. *Soc Indic Res* **159**, 351–377 (2022). https://doi.org/10.1007/s11205-021-02752-1

### Control Variables

We included a number of district-level control variables, such as child mortality, contraceptive prevalence rate (CPR), childlessness, women married below 18 years of age, women workforce participation, women having schooling up to secondary level, exposure to media, poor households, scheduled castes or tribes (SC or ST), Hindu, Muslim, Sikh,and  urban residence in the regression models. The definitions of the control variables are given in Table 1. District-level child mortality was obtained from Guilmoto and Rajan (2013). We estimated CPR, women married below 18 years of age, women having schooling up to secondary level or more, and exposure to media for the 640 districts of India using the NFHS-4. We estimated rest of the control variables from the 2011 census.

### Statistical Analysis

First, we generated scatter-plots to examine unadjusted association of the dependent variables with the independent variables. Then, we estimated Moran’s I to evaluate the degree of spatial clustering in the dependent and independent variables. Moran’s I is a global indicator of spatial autocorrelation and measures the degree to which data points are similar or dissimilar to their spatial neighbours. Moran’s I ranges between −1 and + 1; negative and positive values indicate that closely associated points are more dissimilar or similar, respectively.

We estimated bivariate LISA to examine the local correlation between the dependent variable and the weighted average of the independent variable in the neighbourhood. The bivariate LISA indicates whether the spatial distribution of the independent and the dependent variables are inter-related. The bivariate LISA produce high-high, low-low, high-low, and low–high clusters. For example, high-high IPI and excess female child mortality indicates the districts that have above-average IPI values and above-average excess female child mortality. Likewise, low-low IPI and excess female child mortality indicates the districts that have below-average IPI values and below-average excess female child mortality. While high-high and low-low are known as spatial clusters, high-low and low–high are known as spatial outliers (Anselin, 1995).

We estimated ordinary least squares (OLS) regressions to examine the association of IPI with the dependent variable, net of the control variables. While OLS is a well-accepted tool for understanding associations, OLS estimates are biased when the dependent variables are spatially clustered (Anselin, 2005). Ignoring spatial clustering may be a problem as more variables become significant because standard errors appear smaller than they really are. To account for the spatial correlations, we estimated spatial error regression (SER). SER evaluates spatial clustering of the dependent variable that is not explained by the independent and control variables (Anselin, 2005). SER models account for the unobserved heterogeneity above and beyond the independent variables included in the model. We also estimated spatial autocorrelation coefficients which indicate the fitness of SER models. Finally, we estimated SER models to examine the independent association of 5 dimensions of the IPI with the two outcomes. We used queen’s weight matrix for estimating Moran’s I, LISA and spatial regression.

All variables were adjusted by survey sampling weights provided with NFHS-4. The spatial analysis was conducted in ArcGIS and GeodaSpace; all other analyses were conducted using STATA 16.

## Results

### Descriptive Results

The average IPI value at the district-level was 30.6. IPI ranged between 14.5 (Jaintia Hills district in Meghalaya) and 36.6 (Gaya district in Bihar) (Table 2). The average TFR was 3.3 children per women; the TFR ranged between as low as 1.6 (Kolkata district in West Bengal) and 5.8 (Araria district in Bihar). The average excess female child mortality was 15.8 per 1,000 live births. Excess female child mortality ranged between −17.2 per 1,000 live births (South Andaman district in Andaman and Nicobar Island) and 52.8 per 1,000 live births (Morena district in Madhya Pradesh).

**Fig. 1:**
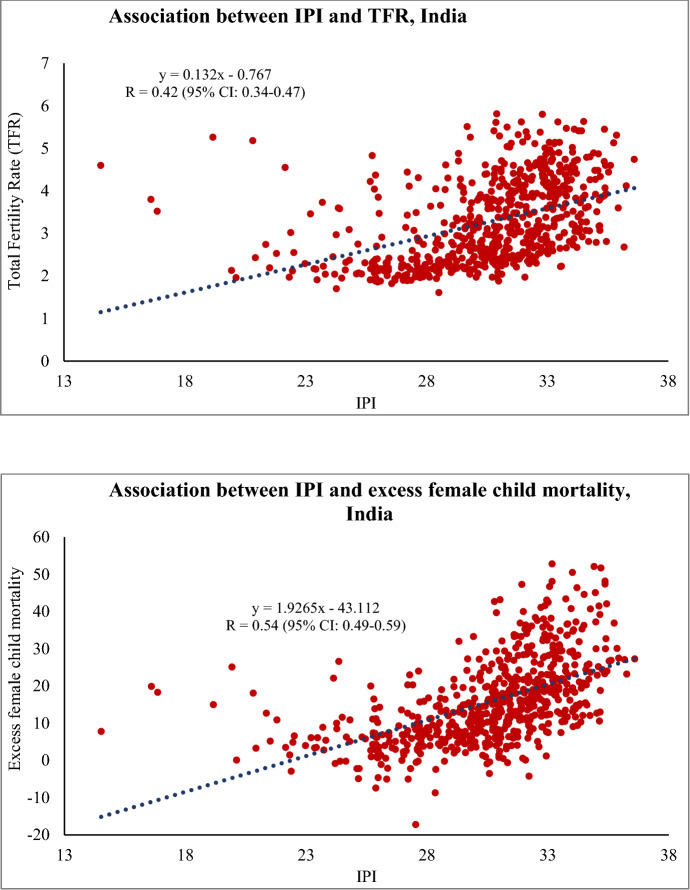
Scatterplots showing unadjusted association of IPI with TFR and excess female child mortality, India

Scatterplots showing unadjusted linear relationship between IPI and the dependent variables are shown in Fig. 1. There was a strong positive association between IPI and excess female child mortality at the district-level (r = 0.54, 95% CI: 0.49–0.59). The magnitude of linear relationship between IPI and TFR was relatively weak (r = 0.42, 95% CI: 0.34–0.47).

### Spatial Clustering of IPI, TFR and Excess Female Child Mortality

There was strong spatial clustering in the IPI across the districts of India; the Moran’s I value was 0.57 (Table 1). Moran’s I values also indicate high spatial clustering in the two dependent variables (0.55 for TFR and 0.59 for excess female child mortality).

**Fig. 2:**
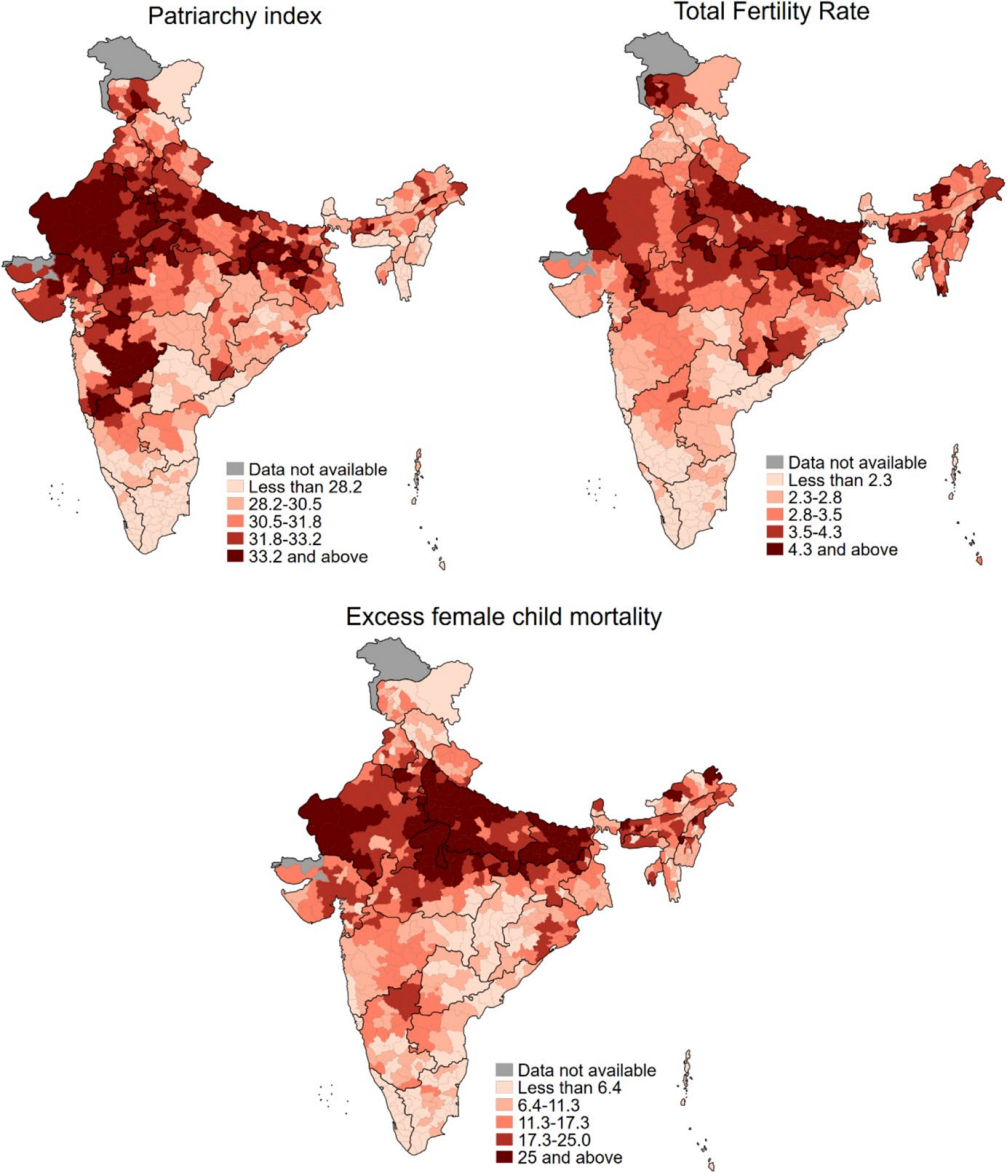
Quintile maps showing spatial distribution of independent and dependent variables, India

Spatial distribution of the IPI, TFR, and excess female child mortality are shown in Fig. 2. High IPI values were clustered primarily in the districts of Haryana, Rajasthan, Uttar Pradesh, Madhya Pradesh, Bihar, Gujarat, and Maharashtra. In contrast, low IPI values were clustered primarily in the districts of Telangana, Kerala, and Tamil Nadu. High TFRs were clustered in the districts of union territory of Jammu and Kashmir, Rajasthan, Uttar Pradesh, Madhya Pradesh, Bihar, Jharkhand, and Meghalaya. Low TFRs were clustered in the south Indian states of Telangana, Andhra Pradesh, Kerala, and Tamil Nadu. Likewise, high excess female child mortality was clustered primarily in the districts of Haryana, Rajasthan, Uttar Pradesh, Madhya Pradesh, Bihar, and Jharkhand, while low excess female child mortality was clustered primarily in the districts of Chhattisgarh, Kerala, and Tamil Nadu. A state and regional labelled locator map of India is shown in "Appendix 1".

**Fig. 3:**
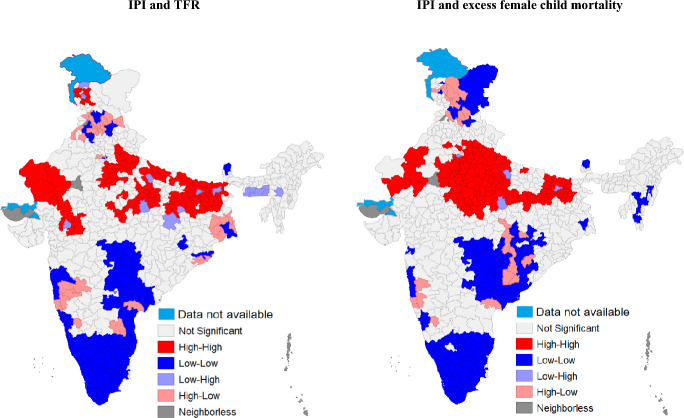
LISA maps showing spatial association of IPI with TFR and excess female child mortality, India

We estimated bivariate LISA to examine the local correlation between the dependent variables and the weighted average of the district-level IPI in the neighborhood (Fig. 3). Clusters of high IPI values and high TFR were located in the northern, central, and eastern India. In particular, such clusters were located in districts of Jhelam valley and outer hills of Jammu and Kashmir, southern and western regions of Rajasthan, central, eastern and southern upper Ganga plain regions of Uttar Pradesh, Bihar, Hazaribagh plateau of Jharkhand, and Vindhya and Malwa regions of Madhya Pradesh. In contrast, clusters of low IPI values and low TFR were located in the western and southern India. In particular, such clusters were located in districts of central plain and southern plain of West Bengal, eastern and inland eastern regions of Maharashtra, Telangana, coastal southern region of Andhra Pradesh, coastal and ghats, inland eastern and inland southern regions of Karnataka, Goa, Kerala, Tamil Nadu, and union territory of Puducherry.

Clusters of high IPI values and high excess female child mortality were located in districts from north eastern and western regions of Rajasthan, central, eastern, northern upper Ganga plain, southern and southern upper Ganga plain regions of Uttar Pradesh, northern and Vindhya regions of Madhya Pradesh, and Bihar. Clusters of low IPI values and low excess female child mortality were located in districts from Jhelam valley and Ladakh regions of Jammu and Kashmir, Mahanadi basin and southern regions of Chhattisgarh, Southern region of Odisha, eastern and inland eastern regions of Maharashtra, inland north eastern region of Telangana, coastal northern region of Andhra Pradesh, inland southern region of Karnataka, Kerala, Tamil Nadu, and union territory of Puducherry.

### OLS Results

IPI was positively associated with TFR and excess female child mortality at district-level, net of other socio-economic, demographic, and residence-related characteristics (Tables 3 and 4). A one unit increase in IPI was associated with a 0.057 unit increase in TFR and 0.854 unit increase in excess female child mortality.

**Table 3:** Results of OLS and SER for TFR, Indian districts

	Ordinary least square (OLS)	Spatial error regression (SER)
Variables	Coefficient (SE)	Coefficient (SE)
IPI	0.057(0.009)**	0.056(0.009)**
Child mortality rate	0.009(0.001)**	0.010(0.001)**
Contraceptive prevalence rate	− 0.011(0.001)**	− 0.008(0.001)**
Childlessness	− 0.032(0.009)**	− 0.017(0.009)
Female workforce participation	− 0.006(0.002)**	− 0.004(0.002)
Female secondary or higher education	− 0.020(0.003)**	− 0.017(0.003)**
Exposure to media	− 0.015(0.002)**	− 0.010(0.002)**
Poor household	0.002(0.002)	0.008(0.002)**
Scheduled caste/tribe	0.002(0.001)	0.002(0.001)**
Hindu	− 0.009(0.001)**	− 0.009(0.001)
Muslim	0.002(0.002)	0.002(0.002)
Sikh	− 0.009(0.002) **	− 0.009(0.003) **
Urban	0.000(0.001)	− 0.001(0.001)
Adjusted R-squared / pseudo R-Squared	77.8	77.1
AIC	840.4	728.3
Spatial autocorrelation (Lambda)		0.532(0.039)
Number of observations	640	640

*p ≤ 0.05, ** *p* < 0.01; Values in the parenthesis are standard errors

**Table 4:** Results of OLS and SER for excess female child mortality, Indian districts

Variables	Ordinary least square (OLS)	Spatial error regression (SER)
	Coefficient (SE)	Coefficient (SE)
IPI	0.854(0.135)**	0.771(0.143)**
TFR	6.666(0.597)**	6.095(0.619)**
Female workforce participation	− 0.193(0.031)**	− 0.164(0.032)**
Female secondary or higher education	− 0.168(0.044)**	− 0.175(0.047)**
Exposure to media	0.025(0.033)	0.04(0.033)
Poor household	− 0.068(0.03)*	− 0.038(0.033)
Scheduled caste/tribe	− 0.077(0.022)**	− 0.066(0.023) **
Hindu	− 0.063(0.022)**	− 0.057(0.024)*
Muslim	− 0.214(0.029)**	− 0.189(0.031)**
Sikh	− 0.068(0.036)	− 0.071(0.041)
Urban	0.085(0.018)**	0.079(0.018)**
Adjusted R-squared / pseudo R-Squared	56.5	57.0
AIC	4400.9	4346.4
Spatial autocorrelation (Lambda)		0.353(0.046)
Number of observations	640	640

*p ≤ 0.05, ** *p* < 0.01; Values in the parenthesis are standard errors

### SER Results

Results from SER models confirm the positive association of IPI with TFR and excess female child mortality at the district level (Tables 3 and 4). In SER, a one unit increase in IPI was associated with 0.056 unit increase in TFR and a 0.771 unit increase in excess female child mortality. The spatial autocorrelation coefficient was statistically significant across all two models, indicating the suitability of SER for modelling these relationships.

The SER results confirm the associations of child mortality with TFR. Child mortality was positively associated with TFR in both the models. While poor households and Scheduled Caste/Tribe were positively associated with TFR, contraceptive prevalence rate, female secondary education, exposure to media, and Sikh were negatively associated.

The SER results confirm the associations of TFR with excess female child mortality. TFR was positively associated with excess female child mortality in the SER. While women workforce participation, female secondary education, scheduled castes/tribes, Hindu, and Muslim were negatively associated with excess female child mortality, urban residence was positively associated.

### Secondary Analyses Results

Of the five dimensions of IPI, only two domains—son preference and socioeconomic domination—were positively associated with TFR. Whereas, domination of men over women and son preference were positively associated with excess female child mortality, i.e. an increase in domination of men over women and son preference were associated with an increase in excess female child mortality, net of other control variables (Table 5). The spatial autocorrelation coefficient was statistically significant across all the models, indicating the suitability of SER for modelling these relationships.

**Table 5:** Results of SER estimating associations of the five dimensions# of patriarchy and the two dependent variables, Indian districts

Variables	TFR	Excess female child mortality
Domination of men over women	0.007(0.026)	1.953(0.423)**
Domination of older generation over the younger generation	0.024(0.020)	0.497(0.336)
Patrilocality	0.021(0.002)	0.462(0.316)
Son preference	0.143(0.025)**	1.628(0.440)**
Socio-economic domination	0.084(0.022)**	0.054(0.382)
Pseudo R-Squared	79.1	58.5
AIC	718.3	4340.6
Spatial autocorrelation (Lambda)	0.470(0.041)**	0.341 (0.047)**
Number of observations	640	640

*p ≤ 0.05, **p < 0.01; Values in the parenthesis are standard errors; All specifications include the full set of controls

^#^Higher values on the five dimensions depict higher patriarchal norms

## Discussion

While past studies used measures, such as female literacy (or sex ratio of literacy rate), number of sons as opposed to daughters, higher ideal proportion of sons, sex ratio at birth; the sex ratio of mortality, proportion of females aged 15–19 unmarried, female labor force participation (or sex ratio of labor force participation), excess female migration, etc. (Arokiasamy, 2002; Bhattacharya, 2006; Clark, 2000; Guilmoto et al., 2018; Malhotra et al., 1995), for examining association of gender inequality with fertility and excess female child mortality in India, our study examines associations of a context-specific index of family patriarchy (IPI) with TFR and excess female child mortality. Results adjusted for key socio-economic, demographic, and residence related characteristics indeed suggest strong associations between IPI and district level TFR and district level excess female child mortality. These results were upheld in the spatial models that accounted for spatial clustering in the two dependent variables. By using a context specific index of patriarchy, we were able to overcome the limitations of previous studies that relied on a few aspects of patriarchy to measure gender inequality.

Our findings extend those of Malhotra et al. (1995) who, examining data from over 40 years ago, concluded that patriarchy plays an important role in explaining regional variations in fertility in India, demonstrating the same findings hold true today. Using a single index of patriarchy, as opposed to multiple indicators (as used in Malhotra et al. (1995)), affords greater opportunity to understand the association of IPI with fertility and excess female child mortality at the district level. Our findings indicate that the association between patriarchy and the two outcomes remains a concern in India, corresponding with other research that documents associations between gender inequality and female disadvantage in child survival (Arokiasamy, 2002; Bhattacharya, 2006; Chaudhuri, 2012, 2015; Clark, 2000; Kashyap, 2019; Kashyap & Behrman, 2020).

This study is also the first to examine how spatial distribution of IPI and spatial distribution of the two outcomes are correlated at the district level. While spatial patterns largely confirmed the north–south divide as identified by Dyson and Moore (1983) 40 years ago, there was widespread spatial heterogeneity in the two outcomes within the north and the south Indian districts. These findings indicate that state-level analysis often masks such district-level spatial heterogeneity and reaffirms the Indian government’s approach to focus on high-need districts and not just high need states.

At the district level, spatial heterogeneity beyond the north–south divide was also evident in the spatial association of IPI with excess female child mortality and TFR. For example, in the northern state of Madhya Pradesh, spatial clusters of high IPI and high excess female child mortality were found only in two (northern and Vindhya regions) of the six geographic regions (Vindhya, central, Malwa, south, south western, and northern). In the south Indian state of Andhra Pradesh, low-low spatial clusters were found only in the one (coastal northern region) of the three regions (coastal northern, coastal southern, and inland southern). Likewise, in Karnataka (a south Indian state), low-low spatial clusters were found only in the one (inland southern region) of the four regions (coastal and ghats, inland eastern, inland southern, and inland northern). District-level spatial heterogeneity in the associations were also noted in TFR within the north and south Indian states. Our findings suggest that spatial analysis can provide more focused inputs to the policy-makers for formulating targeted policy interventions at lower administrative levels, such as districts.

A new and significant contribution of our paper is the use of spatial models for identifying associations of IPI with the two outcomes at the district-level. We were able to identify spatial clusters below the state-level that may require targeted interventions. A few of these spatial clusters comprised of districts across state borders, which may require joint intervention by the states. Spatial models also helped us correctly identify the variables that were associated with the two outcomes. For example, childlessness, female workforce participation and Hindu were negatively associated with fertility in the OLS, but were not statistically significant in the SER. Similarly, poor households was associated with excess female child mortality in the OLS, but lost its significance in the SER. The spatial models confirmed the presence of strong unobserved spatial factors related to local context that may not be captured by the variables included in the study. Therefore, future studies may look for more local factors, such as local cultural practices and local gender discourse, while trying to explain female disadvantage at the district or lower administrative levels.

Another important contribution of our paper is the identification of the dimensions of IPI that were associated with the two outcomes. While domination of men over women and son preference were positively associated with excess female child mortality, son preference and socio-economic domination were associated with TFR. Our findings seem plausible given that in societies where men dominate women, unequal allocation of food, care, and other household resources may lead to female demographic disadvantage (Mishra et al., 2004; Pande, 2003; Pande & Yazbeck, 2003). Guilmoto et al. (2018) also reported association between son preference and excess female child mortality in India. A number of earlier Indian studies have associated son preference with fertility (Arokiasamy, 2002; Bhattacharya, 2006; Clark, 2000; Chaudhuri, 2012; Das Gupta 2003). Our findings are consistent with Clark (2000) who found that women who are disadvantaged in terms of schooling not only want but also attain a higher proportion of sons. Likewise, educated women were less likely to desire more ideal number of sons than daughters in a study by Pande and Astone (2007).

Two dimensions of IPI that are associated with fertility and excess female child mortality and that can be addressed by policy interventions are son preference and socio-economic domination. The Government of India has launched a number of schemes, such as Beti Bachao Beti Padhao and Sukanya Samriddhi Yojana, to promote the value of female children. Similarly, Government of India has launched several schemes, such as Mudra Yojana, Mahila Samriddhi Yojana, Mahila Shakti Kendra, Stand-up India, etc., to encourage women entrepreneurs in India. While these schemes are great initiatives, more needs to be done to overcome challenges related to son preference, women’s work participation and entrepreneurship. Interventions that increase the social, economic and religious utility of women may help in reducing son preference. As there are considerable district-level heterogeneity, policies that account for local-context are likely to be more beneficial for reducing son preference than centrally or state-funded schemes. When it comes to women’s work participation and entrepreneurship, low risk-taking ability, family responsibilities, safety and security are some of the important challenges. Policies that address some of these issues may help in improving the economic value of women in the Indian society.

A key limitation of our study is that we could not replicate this analysis on a more recent dataset—NFHS 2019–21 (henceforth referred to as NFHS-5). The number of districts in NFHS-5 increased to 707 from 640 in NFHS-4. The two outcomes included in our study were estimated using the 2011 Indian census, which included only 640 districts. Since the 2021 Indian census could not be conducted due to COVID-19 pandemic, it is not possible to estimate the excess female child mortality or TFR for all the 707 districts canvassed in NFHS-5. However, while recognizing the importance of this limitation, we believe that social constructs such as patriarchy, and their associations with measures of female disadvantage, are unlikely to substantially change over only in 4–5 years. We were able to demonstrate that patriarchy is important for explaining spatial variations in TFR and excess female child mortality using a large population representative household survey. Future work may consider examining the association of patriarchy with fertility, child mortality, and other demographic indicators at individual level. Third, IPI is specific to kinship structure and should not be generalized to aspects of patriarchy outside of families or households, such as political participation and leadership. Moreover, Singh et al. (2021) did not include ‘lateral relatives’ in the construction of IPI. It may be interesting to examine how the ranking of districts change after inclusion of ‘lateral relatives’ in the patriarchy index. It may also be interesting to examine whether the association between patriarchy index created by including ‘lateral relatives’ and our two outcome variables change at lower administrative levels. Nevertheless, IPI is an additional tool that may be used to assess aspects and trends of gender inequality in LMICs.

Our study also opens up avenues for public health and other social scientists to explore the relationships between patriarchy and challenging public health problems such as physical and sexual violence against women. Likewise, analyzing the associations between IPI and indicators of women’s empowerment may offer an important lens into program or policy levers that could catalyze change to improve the status and value of women in India.

To conclude, IPI was positively associated with TFR and excess female child mortality at the district-level. As patriarchy is deep-rooted in Indian society, a great deal of effort is needed to shift these traditionally held social norms and practices. Some potential interventions in this direction could be redesigning the course curriculum for young children to promote gender equality, further supporting girl child schooling, developing social media campaigns for improving the value of girl child, etc. The use of spatial tools such as LISA to identify subnational clusters where progress is lagging offers valuable planning aids to policy-makers in formulating and implementing targeted interventions at lower administrative levels. This is particularly important given that India is a large and diverse country, and policies formulated at National- or State-levels may not gain the traction and buy-in that more localized programs can find. This granular sub-state analysis also enables the identification of distinct administrative units that may require specially tailored programs and policies reflective of their distinct characteristics and attributes.

## Figures and Tables

**Fig. 4:**
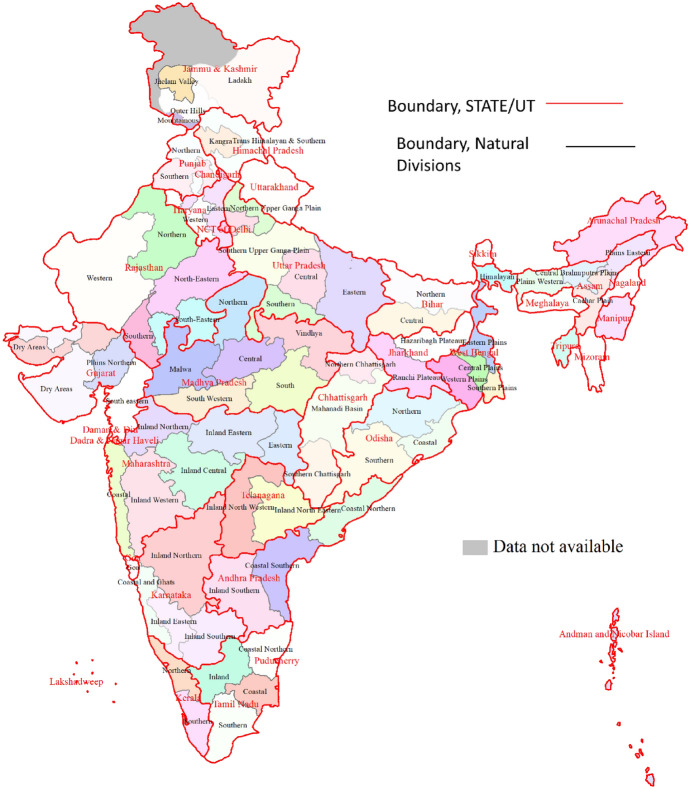
State map with natural divisions within states of India 2017

## Data Availability

The data utilized in this study are available in public domain. The NFHS-4 data can be obtained on request from the Demographic and Health Surveys (DHS) website https://dhsprogram.com/methodology/survey/survey-display-355.cfm. The Census 2011 data may be downloaded from the website ttps://censusindia.gov.in/census.website.

## Appendix 1

See Figure 4.

## Footnotes

1 The India Patriarchy Index is described more fully in Singh et al (2021) and is an adaptation of the Patriarchy Index developed and applied to Europe by Gruber and Szoltysek (2016) and Szoltysek et al. (2017).  In Singh et al (2021) we cite this foundational research.
